# Theoretical and Experimental Study on the Effective Piezoelectric Properties of 1-3 Type Cement-Based Piezoelectric Composites

**DOI:** 10.3390/ma11091698

**Published:** 2018-09-13

**Authors:** Jun Zhu, Zhi Wang, Xingyi Zhu, Bo Yang, Chuanqing Fu

**Affiliations:** 1 College of Mechanical Engineering, Zhejiang University of Technology, Hangzhou 310014, China; zhujun@zjut.edu.cn; 2 Department of Civil Engineering, Zhejiang University, Hangzhou 310058, China; wzhl_2002@163.com; 3 Key Laboratory of Road and Traffic Engineering of Ministry of Education, Tongji University, Shanghai 200092, China; zhuxingyi66@tongji.edu.cn; 4 Department of Civil Engineering, Zhejiang Sci-Tech University, Hangzhou 310018, China; bo.young@163.com; 5 College of Civil Engineering and Architecture, Zhejiang University of Technology, Hangzhou 310014, China

**Keywords:** effective piezoelectric properties, theoretical prediction, experimental study, 1-3 type cement-based piezoelectric composites, double asymptotic homogenization method

## Abstract

The double asymptotic homogenization method originated for analyzing physical systems containing two or more length scales was adopted to predict the characteristic of 1-3 type cement-based piezoelectric composites for the first time. The piezoelectric properties of 1-3 type cement-based piezoelectric composites were measured and comparisons between the experimental data and predicted values validate the effectiveness of the present analytical model. Moreover, numerical discussions and experiments show that one should choose proper volume fraction of constituents to achieve the best performance of the 1-3 type cement-based piezoelectric composites.

## 1. Introduction

Establishment of structural health monitoring (SHM) for important civil infrastructure is considered as an effective tool to ensure the safety, integrity and durability of the structures during their service life [1]. Inspired from the successful experiences in the field of mechanical engineering and space structures, the application of piezoelectric intelligent systems in SHM for civil engineering structures with higher efficiency and lower cost has been the research focus in recent decades [1,2,3,4,5,6,7,8,9]. However, as the key component of intelligent monitoring systems, the conventional smart sensors or actuators originated from aerospace and mechanical engineering are not applicable for concrete structures mainly due to material compatibility problems [10]. Therefore, the concept of cement-based piezoelectric materials which have good interfacial compatibility and acoustic impedance to match the concrete materials is then proposed and regarded as novel functional materials for smart sensor fabrication in civil engineering. 

Li et al. [11] first developed a 0-3 connectivity cement based piezoelectric composite using normal mixing and spread approaches, and the effect of the piezoelectric ceramic volume fraction on the acoustic impedance compatibility between the composite and concrete materials was discussed. After that, Huang et al. [12] fabricated the 0-3 sulfoaluminate cement-based piezoelectric composites and investigated the dominant factors influencing overall behavior of the composites. Chaipanich et al. [13,14] further studied the effect of forming pressure on properties of cement-based piezoelectric composites, where the piezoelectric coefficients and dielectric constants were found to increase with the pressure growth, while the electromechanical coupling coefficient was not sensitive to the pressure changes. To obtain better performance and meet the requirements of engineering utility, 2-2 type cement-based piezoelectric composites were proposed. Li et al. [15] prepared cement-based 2-2 type piezoelectric composites by casting cement-based mortar into a series of pre-arranged piezoelectric thin plates, and the electromechanical and mechanical properties of the composites were then tested. Zhang et al. [16] investigated the actuator effect of the cement-based 2-2 type piezoelectric composites under free and pre-compressed boundary conditions. The influence of the polarization direction of piezoelectric layers on both static and dynamic response of cement-based 2-2 type piezoelectric composites under different loads was studied by Shi’s group [17,18,19].

As compared with other biphasic piezoelectric composites, the 1-3 type cement-based piezoelectric composites with even better piezoelectric, electromechanical and mechanical properties have received much research attention recently. By the dice-and-fill technique, Lam and Chan [20] fabricated the 1-3 type PZT (Lead Zirconate Titanate)-cement composites and investigated the effect of volume fractions of PZT on the electromechanical coupling coefficient. Li et al. [21] prepared and tested the 1-3 type cement-based composites with PMN (Lead Magnesium Niobate) as inclusion, the experimental results indicated that piezoelectric voltage constant $g_{33}$ of the composites was much higher than those of pure PMN, and the acoustic impedance of the composites could be tailored to match the concrete materials. After that, the composites were embedded into concrete beams as the transducers, and the active as well as passive detecting functions of the damage evolution were investigated [22]. Cheng et al. [23] studied the influence of the shape of the piezoelectric inclusion as well as environmental temperature on the effective piezoelectric and dielectric properties of the 1-3 type piezoelectric ceramic-cement composite. Potong et al. [24,25] reported research work on the 1-3 type BZT (Barium Zirconate Titanate)-Portland cement composites, in which the variation of overall dielectric, piezoelectric and hysteresis properties with respect to different parameters was discussed in detail.

In conjunction with the experimental studies, the theoretical prediction of the effective electromechanical behavior of cement-based piezoelectric composites also plays a prominent part in the research. Actually, there exist several methods for predicting the effective properties of piezoelectric composites. Among these approaches, the famous parallel and series models, due to the concise form, have been widely adopted to calculate the effective material constants, especially for the cement based piezoelectric composites [25,26,27]. However, this method can only provide the lower and upper bounds for the effective property estimation. Another one is generally known as the effective medium approach, originated from Eshelby’s classic study [28], which includes the dilute method [29], the Mori–Tanaka method [30], the differential scheme [31], the self-consistent method [32], and the generalized self-consistent method [33]. By generalizing the Mori–Tanaka and Self-consistent approaches, Odegard [34] proposed a new modeling approach to estimate the mechanical properties of piezoelectric composites with better accuracy and convenience. These analytical micromechanical methods allow us to predict multi-axial properties and responses of heterogeneous materials in principle, but usually are used to deal with the particle reinforced composites (e.g., 0-3 piezoelectric composite). Besides the two methods mentioned above, homogenization theory is also a powerful tool to study the global responses of the composites, including the effective material constants, overall stains/stresses, displacements, etc. As for 2-2 type piezoelectric composites, Grekov et al. [35] and Benveniste and Dvorak [36] derived the effective properties based on the hypothesis of equivalent homogeneity, but in different constitutive forms. Using the same theoretical framework, Ray and Batra [37] investigated the effective piezoelectric and elastic properties of 1-3 type carbon nanotube and piezoelectric fiber reinforced composites. It should be noted that the key idea of such homogenization method is to create a uniform field in heterogeneous media by applying proper boundary conditions. 

On the numerical side, Bennett and Hayward [38] employed the finite element model to simulate the effective behavior of 1-3 type piezoelectric composites hydrophones under the hydrostatic environment, and further gave the design guidelines for 1-3 type piezoelectric composites hydrophones with better performance. A finite element model with a unit-cell approach was adopted for the modeling of 0-3 and 1-3 type composites made of piezoceramic fibers embedded in a soft non-piezoelectric matrix, where the estimated longitudinal and transversal effective piezoelectric constants were deduced and compared with some other analytical and experimental data [39]. Pettermann and Suresh [40] developed a comprehensive unit cell model for studying piezoelectric composites with periodic hexagonal or square arrangements of continuous aligned fibers, in which any arbitrary combination of mechanical and electrical loading could be included. Based on a comprehensive finite element numerical model, Kar-Gupta and Venkatesh [41] investigated the effects of variations in the poling characteristics of the matrix and the fiber phase on the overall electromechanical behavior of a 1-3 type piezoelectric composites.

Noticing that the inclusion phase in 1-3 type cement-based piezoelectric composites is usually arranged in a specific pattern with same shape and equal distance, the composites thus can be regarded as a certain periodic structure. Owing to this, in this paper, an alternative homogenization approach called multi-scale asymptotic method is introduced and adopted to estimate the effective properties of 1-3 type cement-based piezoelectric composites. Based on the rigorous mathematical derivation without the skillful chosen of boundary conditions, this method can enable the accurate prediction of both the global and local responses of the composites with periodic inclusions [42,43,44]. Since the inclusions are periodically distributed in a square arrangement in 1-3 type cement-based piezoelectric composites, the expression of the effective properties in explicit forms will be given by the double asymptotic homogenization technique, where the two-scale asymptotic homogenization is implemented twice along two periodic directions, respectively. 

Following the Introduction, the rest of the paper is organized as follows: The preparation of the 1-3 type cement-based piezoelectric composites and the effective piezoelectric coefficients measurement are presented in Section 2. The application of the double asymptotic homogenization method for analytically determining the effective material properties is introduced in detail in Section 3. Section 4 gives the comparison between the analytical solutions and the experimental results; the influence of the volume fraction of PZT inclusion on the effective piezoelectric coefficients is also discussed. Finally, some conclusions are drawn in Section 5 to summarize our main findings. 

## 2. Experiment

**Figure 1:**
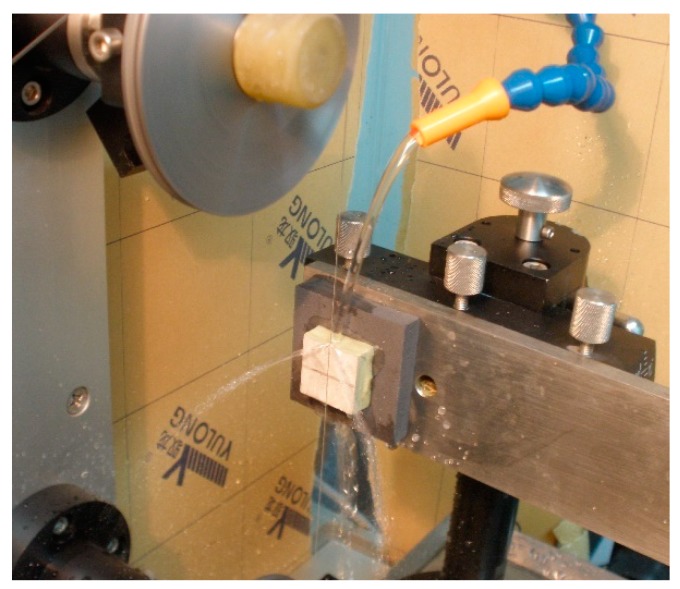
Wire electrical discharge machine.

**Figure 2:**
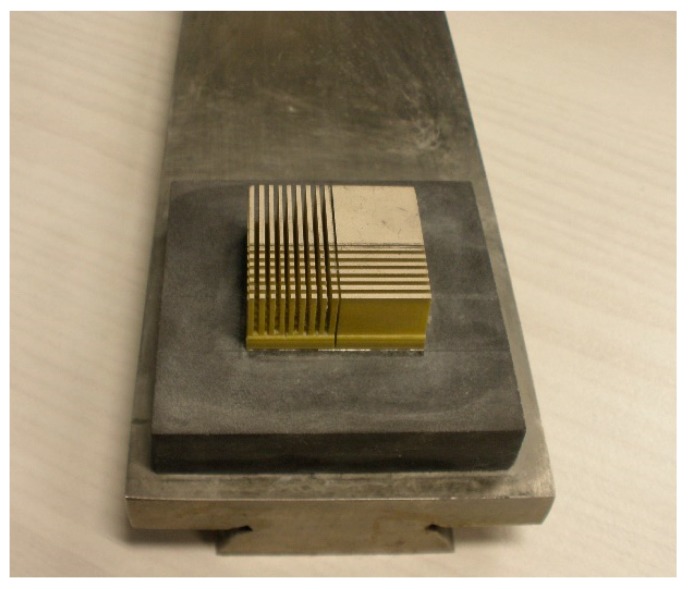
Grooved PZT block.

**Figure 3:**
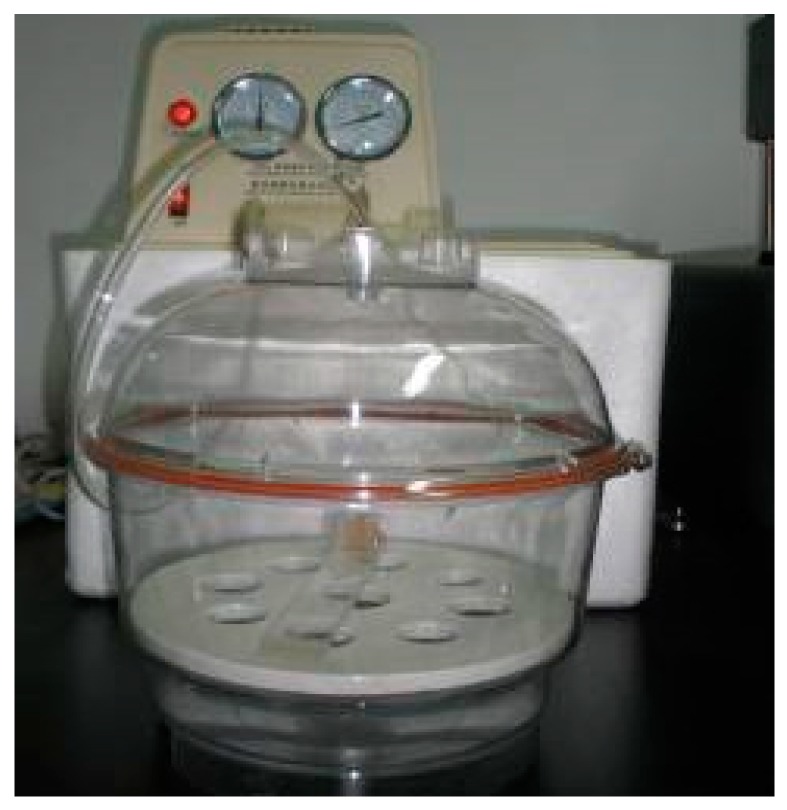
Specimen in a vacuum pump.

**Figure 4:**
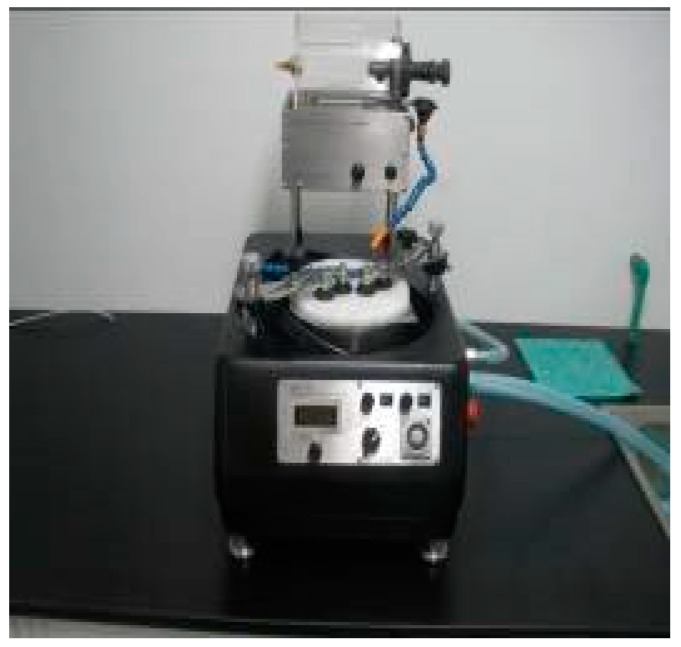
Polishing instrument.

**Figure 5:**
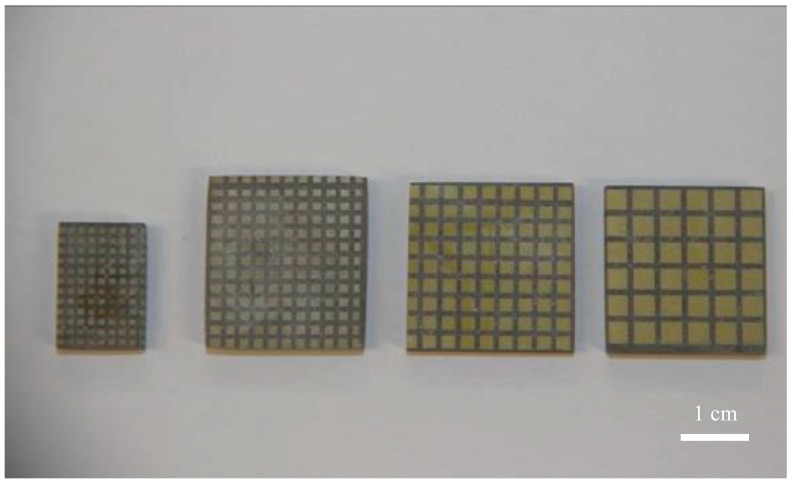
Specimens of 1-3 type cement based PZT composite.

The 1-3 type cement-based piezoelectric composites were prepared using the dice-fill method. The wire electrical discharge machine (WEDM) STX-402 (Figure 1) was used to cut the PZT block (PZT-5H, Baoding Hengsheng Acoustics Electron Apparatus Co., Ltd., Baoding, China) to form the square rods along the polarization direction, where groove spacing was determined according to different volume fraction of constituents. Then, the grooved PZT blocks after cutting (Figure 2) were filled with fresh cement paste with 0.35 cement–water ratio (Portland cement of grade 42.5 from Guangdong First Building Materials Co., Ltd., Guangdong, China), and further placed in vacuum pump (Figure 3) to reduce pores between cement matrix and piezoelectric ceramic rods during casting. The compacted specimens were then cured at 60 °C and 100% relative humidity for 24 h before the gradient drying approach was applied, which includes sequential drying: 60 °C for 4 h, 80 °C for 8 h and 100 °C for 1 h. After polishing the top and bottom surfaces with polishing instrument (Figure 4), the dried specimens were scrubbed with acetone and coated with low-temperature silver paint as electrodes. Figure 5 depicts the specimens with different PZT ceramic volume fractions.

**Figure 6:**
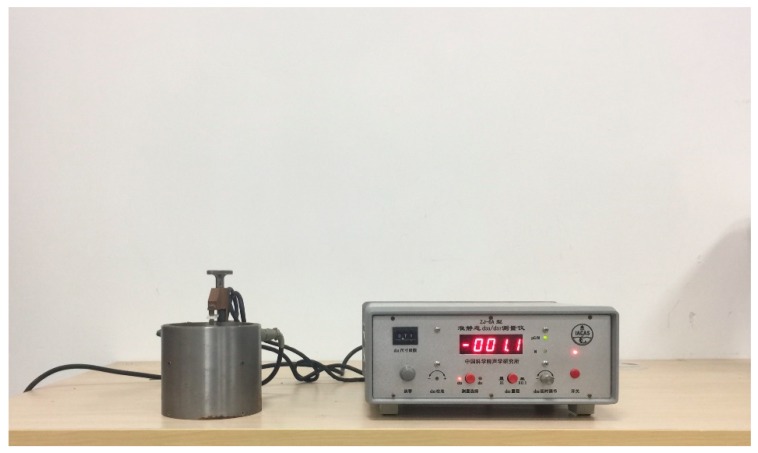
Piezometer.

**Figure 7:**
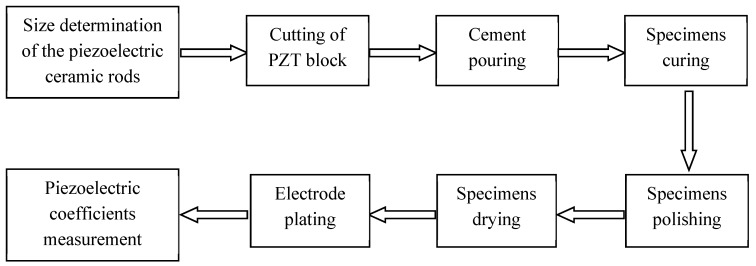
Experiment scheme.

The cement-based piezoelectric composites were aged at room temperature for 24 h prior to the measurement. This study mainly focused on the effective piezoelectric property of the material, therefore only piezoelectric coefficients $d_{31}$, $d_{32}$ and $d_{33}$ of the composites were involved in experiments, and were measured directly by the piezometer (ZJ-6A, Institute of Acoustics Academy of Science, Beijing, China). Figure 6 depicts the piezometer, and the experiment scheme is shown in Figure 7. 

For every volume fraction, the reported experimental data are the average of at least five measurements. The corresponding results are listed in Section 4 for the comparison with the analytical solutions.

## 3. Multiscale Homogenization Model

### 3.1. Basic Equations

Considering transversely isotropic piezoelectric materials, the constitutive relations can be expressed in the Cartesian coordinate system $x_{i}$(i=1,2,3) in a compact form, i.e., Barnett and Lothe notation [45].

(1) $$\sigma_{iJ}=C_{iJMn}u_{M,n}=C_{iJMn}\varepsilon_{Mn}$$

where the subscript comma denotes partial derivative with respect to $x_{i}$. The convention of summation over repeated indices is employed; the generalized displacement vector $u_{I}$, strain tensor $\varepsilon_{Mn}$, stress tensor $\sigma_{iJ}$ and elasticity tensor $C_{iJMn}$ are defined as follows

(2) $$u_{I}=\left\{ \begin{array}{ll} u_{i} & \left(I=i=1,2,3\right)\\ \phi & \left(I=4\right) \end{array}\right.$$

(3) $$\varepsilon_{Mn}=\left\{ \begin{array}{ll} \frac{1}{2}(u_{m,n}+u_{n,m}) & \left(M=m=1,2,3\right)\\ -E_{n}=\phi_{,n} & \left(M=4\right) \end{array}\right.$$

(4) $$\sigma_{iJ}=\left\{ \begin{array}{ll} \sigma_{ij} & \left(J=j=1,2,3\right)\\ D_{j} & \left(J=4\right) \end{array}\right.$$

(5) $$C_{iJMn}=\left\{ \begin{array}{ll} c_{ijmn}^{E} & \left(J=j=1,2,3,M=m=1,2,3\right)\\ e_{nij} & \left(M=4,J=j=1,2,3\right)\\ e_{imn} & \left(J=4,M=m=1,2,3\right)\\ -\kappa_{in}^{\varepsilon } & \left(J=M=4\right) \end{array}\right.$$

where $u_{i}$ and ϕ are the elastic displacements and electrical potential; $\sigma_{ij}$ and $D_{i}$ are the stress and electric displacement components; and $c_{ijmn}^{E}$, $e_{nij}$ and $\kappa_{in}^{\varepsilon }$ are the elastic, piezoelectric and dielectric constants, respectively.

Then, the compact form of the governing equations can be written as

(6) $$\sigma_{iJ,i}=f_{J}$$

where $f_{J}$ are the body force $f_{i}$ (*J* = *i* = 1, 2, 3) and free charge $-\rho_{c}$(J=4).

In addition, the boundary conditions should be specified as

(7) $$\sigma_{iJ}N_{i}=T_{J}^{b}\;(x\in S_{t}\;\mathrm{or}\;x\in S_{d})$$

(8) $$u_{J}=U_{J}^{b}\;(x\in S_{u}\;\mathrm{or}\;x\in S_{\phi })$$

in which $S_{t}$ is the elastic Neumann boundary, $S_{d}$ is the electric Neumann boundary, $S_{u}$ is the elastic Dirichlet boundary, $S_{\phi }$ is the electric Dirichlet boundary, $N_{i}$ (*i* = 1, 2, 3) is the outer unit normal on the boundary, $T_{J}^{b}$ is the generalized external load, and $U_{J}^{b}$ is the generalized displacement.

### 3.2. Basic Theory of Multiscale Homogenization

Consider the piezoelectric composites made of periodic unit cells, where either geometrical or material parameters satisfy the following relation

(9) R (x+NY)=R (x)

where $x\;(x_{1},\;x_{2},\;x_{3})$ is the position vector of the point in the macroscopic scale coordinate, $N=\mathrm{diag}[n_{1},\;n_{2},\;n_{3}]$ with $n_{i}$(*i* = 1, 2, 3) being arbitrary integer numbers, and $Y={\left[Y_{1},\;Y_{2},\;Y_{3}\right]}^{T}$ a constant vector determining the periodicity of the composite. According to the two-scale asymptotic expansion method proposed by Babuška et al. [46,47], let the dimensionless parameter ϑ be defined as the ratio between the characteristic length of the unit cell and that of the entire composite, and introduce the corresponding local coordinate ξ=x/ϑ. Then, the generalized displacement u(x) and stress σ(x) can be expressed in the following two-scale expansion as

(10) $$u(x)=u_{}^{\vartheta }(x,\xi ;\vartheta )=u_{}^{0}(x,\xi ;\vartheta )+\vartheta u_{}^{1}(x,\xi ;\vartheta )+\vartheta^{2}u_{}^{2}(x,\xi ;\vartheta )+\cdots \cdots$$

(11) $$\sigma (x)=\sigma_{}^{\vartheta }(x,\xi ;\vartheta )=\sigma^{0}(x,\xi ;\vartheta )+\vartheta \sigma^{1}(x,\xi ;\vartheta )+\vartheta^{2}\sigma_{}^{2}(x,\xi ;\vartheta )+\cdots \cdots$$

In view of the expression of material constant $C^{\vartheta }(x)=C(x/\vartheta )=C(\xi )$, by substituting Equation (10) into Equation (1), and comparing the terms in Equation (11) with the same power of ϑ, we may obtain the following expressions

(12) $$0=C_{iJMn}^{\vartheta }\frac{\partial u_{M}^{0}}{\partial \xi_{n}}$$

(13) $$\sigma_{iJ}^{0}(x,\xi ;\vartheta )=C_{iJMn}^{\vartheta }(\frac{\partial u_{M}^{0}}{\partial x_{n}}+\frac{\partial u_{M}^{1}}{\partial \xi_{n}})$$

(14) $$\sigma_{iJ}^{1}(x,\xi ;\vartheta )=C_{iJMn}^{\vartheta }(\frac{\partial u_{M}^{1}}{\partial x_{n}}+\frac{\partial u_{M}^{2}}{\partial \xi_{n}})$$

Similarly, substitution of Equation (11) into Equation (6) yields

(15) $$\frac{\partial \sigma_{iJ}^{0}}{\partial \xi_{i}}=0$$

(16) $$\frac{\partial \sigma_{iJ}^{0}}{\partial x_{i}}+\frac{\partial \sigma_{iJ}^{1}}{\partial \xi_{i}}+f_{J}=0$$

It can be observed from Equation (12) that $u_{M}^{0}$ only depends on the macroscopic coordinate *X*, therefore the generalized displacement in Equation (10) can be written as

(17) $$u(x)=u_{}^{\vartheta }(x,\xi ;\vartheta )=u_{}^{0}(x;\vartheta )+\vartheta u_{}^{1}(x,\xi ;\vartheta )+\vartheta^{2}u_{}^{2}(x,\xi ;\vartheta )+\cdots \cdots$$

where $u_{}^{0}(x;\vartheta )$ is the macroscopic displacement, and $u_{}^{1}(x,\xi ;\vartheta )$, $u_{}^{2}(x,\xi ;\vartheta )$, … are the mesoscopic displacements [48], which denote the perturbation displacements of the mesoscopic structure.

Since $\sigma_{iJ}^{1}(x,\xi ;\vartheta )$ are functions with a periodicity of Y, it follows that Equation (16) will have a unique solution if

(18) $$\int_{Y}\left(-\frac{\partial \sigma_{iJ}^{0}}{\partial x_{i}}-f_{J}\right)d\xi =0$$

which can be written as

(19) $$\frac{\partial <\sigma_{iJ}^{0}>}{\partial x_{i}}+f_{J}=0$$

where

(20) $$<\cdot >=\frac{1}{V}\int_{Y}(\cdot )d\xi$$

in which V is the volume of the unit cell. 

Assuming that the displacement $u_{}^{1}(x,\xi ;\vartheta )$ takes the form as [49]

(21) $$u_{I}^{1}(x,\xi ;\vartheta )=L_{IJm}(\xi )\frac{\partial u_{J}^{0}(x;\vartheta )}{\partial x_{m}}$$

where $L_{IJm}(\xi )$ with respect to ξ are auxiliary functions with a periodicity of Y. 

Substitution of Equation (21) into Equation (13) and taking the average volume integration over the unit cell yields

(22) $$<\sigma_{iJ}^{0}(x,\xi ;\vartheta )>=C_{iJPq}^{\mathrm{Eff}}\frac{\partial u_{P}^{0}(x;\vartheta )}{\partial x_{q}}$$

where

(23) $$C_{iJPq}^{\mathrm{Eff}}=<C_{iJPq}^{\vartheta }+C_{iJMn}^{\vartheta }\frac{\partial L_{MPq}}{\partial \xi_{n}}>=\frac{1}{V}\int_{Y}(C_{iJPq}^{\vartheta }+C_{iJMn}^{\vartheta }\frac{\partial L_{MPq}}{\partial \xi_{n}})d\xi$$

can be considered as the effective material properties of the composite.

As we can see, the key step to solve the effective material properties lies on the function L(ξ), which usually can be easily determined for the two-phase composites with only one periodic direction (2-2 type composites) [50]. However, for most kinds of 1-3 type composites with reinforced phase periodically distributed in the matrix along two directions, it is very difficult to obtain the analytical expression of the auxiliary function L(ξ), although there is an exception for the cylindrical inclusions where the explicit form of $\partial L_{PMn}/\partial \xi_{q}$ can be solved invoking the potential methods of complex variable and Weierstrass elliptic functions [51]. Of course, complicated numerical implementation through the finite element method (FEM) can also achieve the same goal (see [52] for more details). Therefore, for the 1-3 type cement-based piezoelectric composites with inclusions of square cross-section involved in this paper, the double asymptotic homogenization method which means twice implementation of multiscale homogenization along two periodic directions, is adopted to analytically predict the effective material properties.

### 3.3. First Homogenization for the 1-3 Type Cement Based Piezoelectric Composite

**Figure 8:**
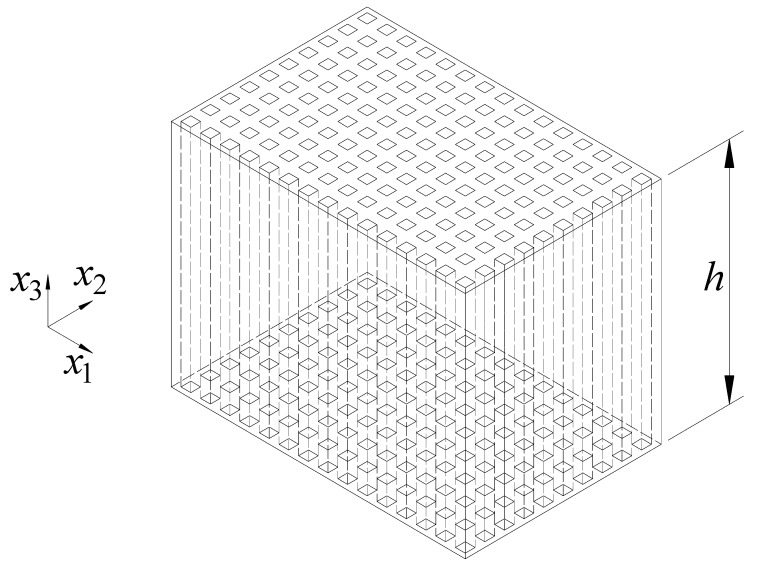
Three-dimensional schematic of the 1-3 type cement-based piezoelectric composite.

**Figure 9:**
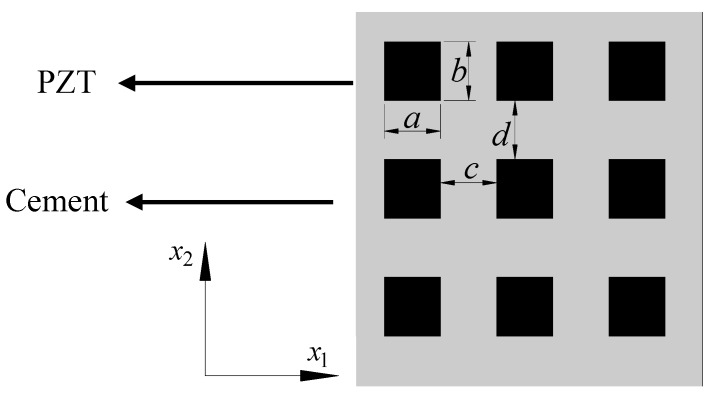
Planar schematic of the 1-3 type cement-based piezoelectric composite.

**Figure 10:**
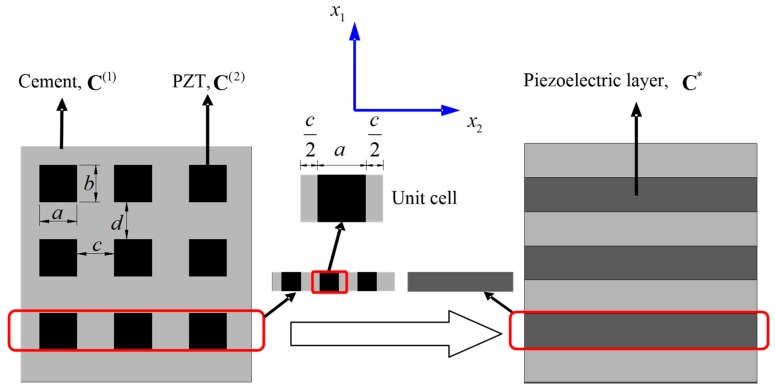
First homogenization of the 1-3 cement-based piezoelectric composite.

Consider the 1-3 type cement-based piezoelectric composite shown in Figure 8 and Figure 9, in which the PZT rods with the polarization direction parallel to $x_{3}$ axis are assumed to array periodically along the $x_{1}$ and $x_{2}$ direction, and the material constants of the cement and PZT are denoted by $C^{\left(1\right)}$ and $C^{\left(2\right)}$, respectively. The cross section perpendicular to $x_{3}$ axis will first be divided into laminated structure composed of alternate stacking of homogenous cement layers and inhomogeneous mixed layers with periodic PZT inclusions, as depicted in Figure 10. After that, the first homogenization will be conducted along $x_{2}$ direction only in the mixed area, where the material properties of the unit cell can be expressed as

(24) $$C^{}=\left\{ \begin{array}{ll} C^{\left(1\right)} & \left(0\leq x_{2}<\frac{1}{2}c\right)\\ C^{\left(2\right)} & \left(\frac{1}{2}c\leq x_{2}<a+\frac{1}{2}c\right)\\ C^{\left(1\right)} & \left(a+\frac{1}{2}c\leq x_{2}\leq \frac{1}{2}c\right) \end{array}\right.$$

in which a is the side length of the PZT cross section along $x_{2}$ direction, while c is the distance between two PZT rods along the same direction, as shown in Figure 10. 

Since each two-phase layer has periodicity only in $x_{2}$ direction, during the first homogenization, the expression of the effective property for the inhomogeneous layer $C_{iJPq}^{*}$ can be reduced to

(25) $$C_{iJPq}^{*}=<C_{iJPq}^{\vartheta }+C_{iJM2}^{\vartheta }\frac{\partial L_{MPq}}{\partial \xi_{2}}>$$

In view of Equations (21) and (13), Equation (15) can be written in the ordinary differential form with respect to $\xi_{2}$ while noticing the fact that $u_{}^{0}(x;\vartheta )$ is independent of the local coordinate ξ, i.e.,

(26) $$\frac{d(C_{2JMn}^{\vartheta }+C_{2JP2}^{\vartheta }\frac{dL_{PMn}}{d\xi_{2}})}{d\xi_{2}}=0$$

Integration of Equation (26) leads to

(27) $$C_{2JMn}^{\vartheta }+C_{2JP2}^{\vartheta }\frac{dL_{PMn}}{d\xi_{2}}=G_{2JMn}$$

which can be further rewritten as

(28) $$L_{PMn,2}=K_{PJ}(G_{2JMn}-C_{2JMn}^{\vartheta })$$

where $G_{2JMn}$ are the unspecified constants and the constant matrix K is the inverse matrix of $C_{2JP2}^{\vartheta }$

(29) $$K_{RJ}C_{2JP2}^{\vartheta }=\delta_{RP}\;(R=1,2,3,4;J=1,2,3,4;P=1,2,3,4)$$

Making use of the periodic condition $L_{PMn}(\xi_{2})=L_{PMn}(\xi_{2}+Y_{2})$, the average volume integration of Equation (28) over the unit cell gives rise to the following equation

(30) $$\begin{array}{ll} 0= & \int_{0}^{0.5c}K_{PJ}^{\left(1\right)}(G_{2JMn}^{}-C_{2JMn}^{\vartheta (1)})d\xi_{2}+\\ & \int_{0.5c}^{a+0.5c}K_{PJ}^{\left(2\right)}(G_{2JMn}^{}-C_{2JMn}^{\vartheta (2)})d\xi_{3}+\int_{a+0.5c}^{a+c}K_{PJ}^{\left(1\right)}(G_{2JMn}^{}-C_{2JMn}^{\vartheta (1)})d\xi_{2} \end{array}$$

where superscripts (1) and (2) denote the cement and PZT phases, respectively. Since the two phases of the composite are assumed to be homogeneous, then the above equation can be rewritten as

(31) $$(cK_{PJ}^{\left(1\right)}+aK_{PJ}^{\left(2\right)})G_{2JMn}^{}=cK_{PJ}^{\left(1\right)}C_{2JMn}^{\vartheta (1)}+aK_{PJ}^{\left(2\right)}C_{2JMn}^{\vartheta (2)}$$

which can be further arranged as

(32) $$G_{2JMn}^{}=Z_{JP}(cK_{PQ}^{\left(1\right)}C_{2QMn}^{\vartheta (1)}+aK_{PQ}^{\left(2\right)}C_{2QMn}^{\vartheta (2)})$$

where the constant matrix Z is the inverse matrix of $cK_{PJ}^{\left(1\right)}+aK_{PJ}^{\left(2\right)}$

(33) $$Z_{RP}(cK_{PJ}^{\left(1\right)}+aK_{PJ}^{\left(2\right)})=\delta_{RJ}\;(R=1,2,3,4;P=1,2,3,4;J=1,2,3,4)$$

By virtue of Equations (25), (28), (29), (32) and (33), the analytical solution of effective property $C_{iJPq}^{*}$ after first homogenization can be finally determined as

(34) $$C_{iJPq}^{*}=<C_{iJPq}^{}>-v_{1}(1-v_{1})T_{MN}R_{MNiJPq}$$

where

(35) $$v_{1}=\frac{a}{a+c}$$

(36) $$\langle C_{iJPq}^{}\rangle =\frac{1}{V}\int_{Y}C_{iJPq}^{}d\xi =v_{1}C_{iJPq}^{\left(2\right)}+(1-v_{1})C_{iJPq}^{\left(1\right)}$$

(37) $$T_{MN}={\left(v_{1}C_{2MN2}^{\left(1\right)}-(1-v_{1})C_{2MN2}^{\left(2\right)}\right)}^{-1}$$

(38) $$R_{MNiJPq}=(C_{iJM2}^{\left(1\right)}-C_{iJM2}^{\left(2\right)})(C_{2NPq}^{\left(1\right)}-C_{2NPq}^{\left(2\right)})$$

For the sake of brevity, the non-zero components of $C_{iJPq}^{*}$ in Voigt notation are listed in Appendix A.

### 3.4. Second Homogenization for the 1-3 Type Cement Based Piezoelectric Composite

**Figure 11:**
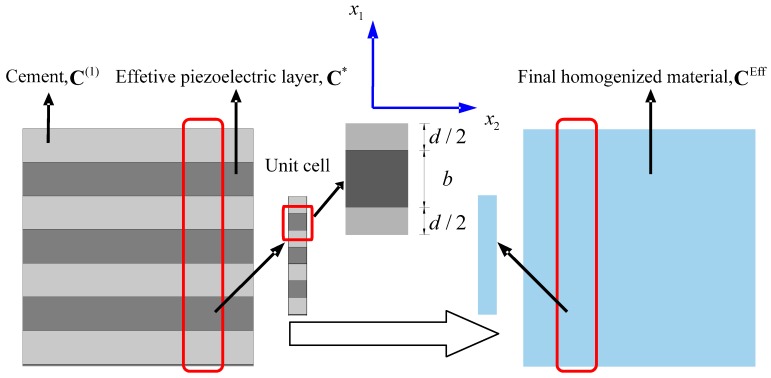
Second homogenization of the 1-3 type cement based piezoelectric composite.

After the first homogenization, the initial 1-3 type cement-based piezoelectric composite has been turned into the 2-2 type composite composed of cement and effective piezoelectric phase whose material properties $C^{*}$ are calculated previously, as shown in Figure 11. Following a similar procedure to that presented in Section 3.3, the second homogenization over the unit cell (Figure 11) along $x_{1}$ direction will give rise to final effective properties of the composite denoted as $C^{\mathrm{Eff}}$, of which the analytical solution can be expressed similar to Equations (34)–(38)

(39) $$C_{iJPq}^{\mathrm{Eff}}=<{\bar{C}}_{iJPq}^{}>-v_{2}(1-v_{2}){\bar{T}}_{MN}{\bar{R}}_{MNiJPq}$$

where

(40) $$v_{2}=\frac{b}{b+d}$$

(41) $$\langle {\bar{C}}_{iJPq}^{}\rangle =\frac{1}{V}\int_{Y}{\bar{C}}_{iJPq}^{}d\xi =v_{2}C_{iJPq}^{*}+(1-v_{2})C_{iJPq}^{\left(1\right)}$$

(42) $${\bar{T}}_{MN}={\left(v_{2}C_{1MN1}^{\left(1\right)}-(1-v_{2})C_{1MN1}^{*}\right)}^{-1}$$

(43) $${\bar{R}}_{MNiJPq}=(C_{iJM1}^{\left(1\right)}-C_{iJM1}^{*})(C_{1NPq}^{\left(1\right)}-C_{1NPq}^{*})$$

As we can see, the actual difference from the previous calculation in Equations (34)–(38) is just the replacement of $C_{iJPq}^{\left(2\right)}$ with $C_{iJPq}^{*}$ and subscript 2 with 1 in property tensor. The non-zero components of $C_{iJPq}^{\mathrm{Eff}}$ in Voigt notation are also listed in Appendix B for brevity.

## 4. Results and Discussion

This section illustrates the comparison between the experimental results and the theoretical prediction of the effective material properties. Besides the described homogenization model, the comparison is also made with other proposed model [53] from the literature. The corresponding material parameters in the calculation are listed in Table 1. Since the electromechanical coupling coefficients calculated by the analytical model are the piezoelectric stress coefficients which cannot be directly compared with the measured piezoelectric strain coefficients, $d_{ij}^{}$, the following transformation should be carried out before comparison

(44) $$d_{ij}^{\mathrm{Eff}}=e_{im}^{\mathrm{Eff}}g_{mj}^{\mathrm{Eff}}\;$$

where $e_{mj}^{\mathrm{Eff}}$ is the effective piezoelectric stress coefficient, and $g_{mj}^{\mathrm{Eff}}$ is the inverse matrix of the elastic constant $c_{mj}^{\mathrm{Eff}}$.

**Figure 12:**
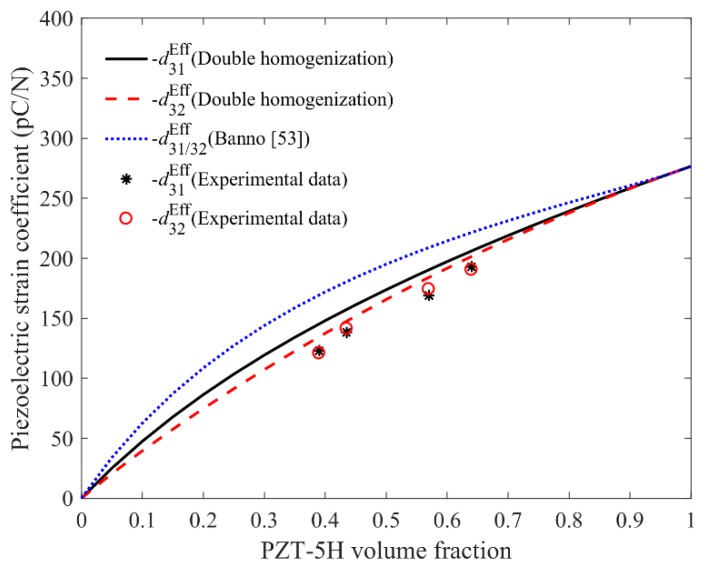
Comparison between the experimental and theoretical values of $d_{31}^{\mathrm{Eff}}$ and $d_{32}^{\mathrm{Eff}}$.

**Figure 13:**
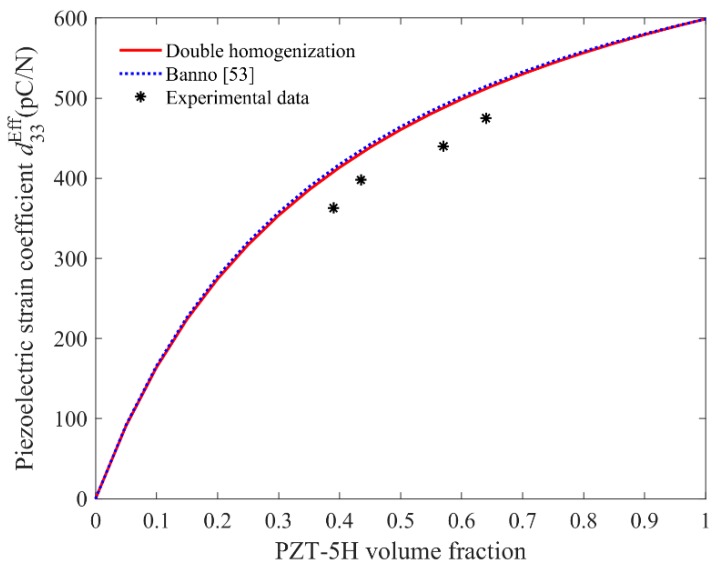
Comparison between the experimental and theoretical values of $d_{33}^{\mathrm{Eff}}$.

Figure 12 and Figure 13 display the influence of volume fraction f ($f=v_{1}v_{2}=ab/[(a+c)(b+d)]$) of PZT on the variation of $d_{31}^{\mathrm{Eff}}$, $d_{32}^{\mathrm{Eff}}$ and $d_{33}^{\mathrm{Eff}}$. In Figure 12, the theoretical curves of $-d_{31}^{\mathrm{Eff}}$ and $-d_{32}^{\mathrm{Eff}}$ obtained by the present approach grow almost linearly with the increase of volume fraction of PZT, and show good agreement with the experimental data. Meanwhile, the dotted line calculated by theoretical model introduced in [53] over predicts the piezoelectric coefficients, thus proves the accuracy of the double homogenization method. Furthermore, one can also notice that the calculated values of $d_{31}^{\mathrm{Eff}}$ and $d_{32}^{\mathrm{Eff}}$ are not exactly the same, as expected, which is mainly due to the adoption of the asymptotic homogenization method along two directions but not at the same time. However, such difference is quite slight, which can be ignored in the practical application.

In Figure 13, the two theoretical curves of piezoelectric coefficient $d_{33}^{\mathrm{Eff}}$ having the same trend show an evident non-linear variation with the change in inclusion volume fraction, while the agreement between theoretical and experimental results is seen to be good. Note that the results obtained from [53] agree remarkably well with the present method, which may correspond to the similar hypotheses concerning the states of stress and strain in the body as in parallel model. 

**Figure 14:**
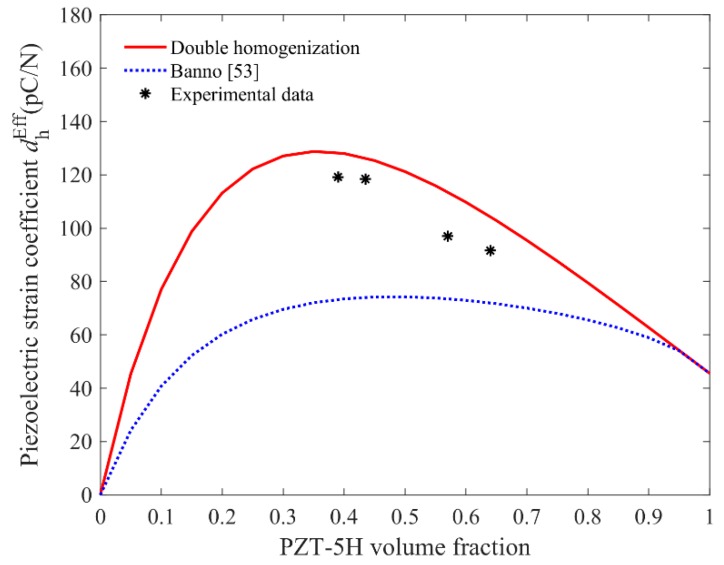
Comparison between the experimental and theoretical values of $d_{h}^{\mathrm{Eff}}$.

In addition to the above-mentioned electromechanical coupling coefficients, hydrostatic charge coefficient $d_{h}^{\mathrm{Eff}}$ which has the definition as $d_{h}^{\mathrm{Eff}}=d_{31}^{\mathrm{Eff}}+d_{32}^{\mathrm{Eff}}+d_{33}^{\mathrm{Eff}}$, is another useful parameter in evaluating piezoelectric materials, especially in the application of hydrophone [54]. Therefore, Figure 14 illustrates the experimental values of $d_{h}^{\mathrm{Eff}}$ as well as the theoretical ones versus different volume fraction of PZT. Due to the difference between the two predicted curves of $d_{31}^{\mathrm{Eff}}$ and $d_{32}^{\mathrm{Eff}}$ observed in Figure 12, the theoretical model from [53] underestimates the hydrostatic charge coefficient, while the present model can matches quite well with the experimental results. The calculated value of $d_{h}^{\mathrm{Eff}}$ grows rapidly at first and then decreases as the volume fraction of PZT increases, where the peak value appears near *f* = 0.35. It also implies that the optimal effective piezoelectric property of the composites depends on the proper volume fraction of the PZT inclusion.

To further analyze the quantitative difference between the calculated and experimental results in Figure 12, Figure 13 and Figure 14, specified data comparison and relative errors for different volume fraction f of PZT are listed in Table 2, where almost the same tested values of $d_{31}^{\mathrm{Eff}}$ and $d_{32}^{\mathrm{Eff}}$ indicate that the homogenized composite is transversely isotropic, which is mainly due to the equal-spaced distribution of the PZT inclusion with square cross section. The possible source of discrepancy observed in Table 2 may correspond to the fact that the characteristic size of the periodic substructure in composite is not small enough and the samples might not be regarded as homogeneous materials by the probe of the piezometer, which would subsequently affect the test results. We believe that the theoretical prediction would match the experiment quantitatively much better if the characteristic size of the composite is reduced.

## 5. Conclusions

In this study, the overall piezoelectric properties of the 1-3 type cement-based piezoelectric composites were studied both experimentally and theoretically. Composites with different volume fraction of PZT inclusion were prepared using the dice-fill approach, and the corresponding piezoelectric strain coefficients were measured directly by the piezometer and subsequently compared with the theoretically estimated results which were calculated through the double asymptotic homogenization method and other analytical models from the literature. Good agreement between the calculated results and the experimental data validates the analytical model adopted in this paper and further parametric studies show how the volume fraction of PZT inclusion affects the variation of the effective piezoelectric properties of the composites. Owing to the explicit formula and accuracy, the double homogenization method is very suitable for the prediction and optimization of the effective properties of the 1-3 cement-based piezoelectric composites.

## Figures and Tables

**Table 1:** Material properties of the considered composites ^a,b^.

Material	Elastic Constant (GPa)					Piezoelectric Coefficient $\left({C/m}^{2}\right)$			Relative Dielectric Constant ^c^			
	$c_{11}^{}$	$c_{12}^{}$	$c_{13}^{}$	$c_{33}^{}$	$c_{44}^{}$	$e_{31}$	$e_{33}$	$e_{15}$	$\kappa_{11}^{\varepsilon }/\kappa_{0}$	$\kappa_{33}^{\varepsilon }/\kappa_{0}$	$\kappa_{11}^{\sigma }/\kappa_{0}$	$\kappa_{33}^{\sigma }/\kappa_{0}$
PZT-5H	127	80.2	84.7	117	23	−6.6	23.2	17	3131	3400	4551	5366
Cement ^d^	15.4	3.9	3.9	15.4	5.8	0	0	0	19	19	19	19

^a^ Voigt notation is used. ^b^ Only the inclusion phase is polarized. ^c^ $\kappa_{0}=8.85\times 10^{-12}(C^{2}{/\mathrm{Nm}}^{2})$ vacuum dielectric constant; $\kappa_{}^{\varepsilon }$ dielectric constant at constant strain; $\kappa_{}^{\sigma }$ dielectric constant in the stress-free state. ^d^ Young’s modulus is 13.9 GPa, Poisson’s ratio is 0.2.

**Table 2:** Comparison between theoretical and experimental values of the piezoelectric strain coefficients.

Effective Piezoelectric Parameters		Sample Number			
		1	2	3	4
**Size parameter (mm)**	*a*	1.24	0.95	2	3
	*b*	1.24	0.95	2	3
	*c*	0.74	0.49	0.65	0.75
	*d*	0.74	0.49	0.65	0.75
	*h* ^e^	6.67	6.47	5.3	5.8
**Volume fraction of PZT *f***		0.39	0.435	0.57	0.64
$-d_{31}^{\mathrm{Eff}}$ **(pC/N)**	*Present model*	145	157	190	206
	*Experimental*	123	138	169	193
	*Relative error* ^f^	0.15	0.12	0.11	0.06
$-d_{32}^{\mathrm{Eff}}$ **(pC/N)**	*Present model*	135	148	184	202
	*Experimental*	121	141	174	190
	*Relative error*	0.10	0.04	0.05	0.06
$d_{33}^{\mathrm{Eff}}$ **(pC/N)**	*Present model*	408	431	488	512
	*Experimental*	363	398	440	475
	*Relative error*	0.11	0.08	0.10	0.07
$d_{h}^{\mathrm{Eff}}$ **(pC/N)**	*Present model*	128	126	114	104
	*Experimental*	119	119	97	92
	*Relative error*	0.07	0.06	0.15	0.12

^e^ Height of the specimen in $x_{3}$ direction (Figure 8); ^f^ Relative errors are defined as |Theoretical − Experimental|/Theoretical.

## Appendix A. Non-Zero Components of the Generalized Elasticity Tensors after the First Homogenization

**(a) Piezoelectric coefficients:**

(A1) $$\begin{array}{l} e_{24}^{*}=\frac{\left(-e_{24}^{\left(2\right)}+e_{24}^{\left(1\right)}\right)\kappa_{22}^{\varepsilon (1)}v_{1}+e_{24}^{\left(1\right)}q_{42}}{q_{42}}\\ e_{32}^{*}=-\frac{\left(e_{32}^{\left(2\right)}-e_{32}^{\left(1\right)}\right)c_{22}^{\left(2\right)}v_{1}+\left(-e_{32}^{\left(2\right)}+e_{32}^{\left(1\right)}\right)c_{22}^{\left(2\right)}-e_{32}^{\left(2\right)}q_{12}}{q_{12}}\\ e_{15}^{*}=e_{15}^{\left(2\right)}v_{1}+e_{15}^{\left(1\right)}-e_{15}^{\left(1\right)}v_{1}\\ e_{33}^{*}=-\frac{v_{1}e_{33}^{\left(1\right)}q_{12}-v_{1}p_{52}-v_{1}e_{33}^{\left(2\right)}q_{12}+v_{1}{}^{2}p_{52}-e_{33}^{\left(1\right)}q_{12}}{q_{12}}\\ e_{31}^{*}=-\frac{-e_{31}^{\left(1\right)}q_{12}+v_{1}{}^{2}p_{62}-v_{1}p_{62}-v_{1}e_{31}^{\left(2\right)}q_{12}+v_{1}e_{31}^{\left(1\right)}q_{12}}{q_{12}} \end{array}$$

**(b) Relative dielectric constants:**

(A2) $$\begin{array}{l} \kappa_{11}^{\varepsilon *}=\kappa_{11}^{\varepsilon (2)}v_{1}+\kappa_{11}^{\varepsilon (1)}-\kappa_{11}^{\varepsilon (1)}v_{1}\\ \kappa_{33}^{\varepsilon *}=\frac{-v_{1}\kappa_{33}^{\varepsilon (1)}q_{12}+v_{1}\kappa_{33}^{\varepsilon (2)}q_{12}-v_{1}{}^{2}p_{72}+\kappa_{33}^{\varepsilon (1)}q_{12}+v_{1}p_{72}}{q_{12}}\\ \kappa_{22}^{\varepsilon *}=-\frac{\kappa_{22}^{\varepsilon (1)}\kappa_{22}^{\varepsilon (2)}}{q_{42}} \end{array}$$

**(c) Elastic constants:**

(A3) $$\begin{array}{l} c_{11}^{*}=-\frac{v_{1}{}^{2}p_{12}+v_{1}(-c_{11}^{\left(2\right)}q_{12}-p_{12}+c_{11}^{\left(1\right)}q_{12})-c_{11}^{\left(1\right)}q_{12}}{q_{12}}\\ c_{12}^{*}=-\frac{(c_{12}^{\left(2\right)}-c_{12}^{\left(1\right)})c_{22}^{\left(2\right)}\left(v_{1}-1\right)-c_{12}^{\left(2\right)}q_{12}}{q_{12}}\\ c_{13}^{*}=-\frac{v_{1}{}^{2}p_{22}+v_{1}(-c_{13}^{\left(2\right)}q_{12}-p_{22}+c_{13}^{\left(1\right)}q_{12})-c_{13}^{\left(1\right)}q_{12}}{q_{12}}\\ c_{22}^{*}=-\frac{c_{22}^{\left(1\right)}c_{22}^{\left(2\right)}}{q_{12}}\\ c_{23}^{*}=-\frac{(c_{23}^{\left(2\right)}-c_{23}^{\left(1\right)})c_{22}^{\left(2\right)}\left(v_{1}-1\right)-c_{23}^{\left(2\right)}q_{12}}{q_{12}}\\ c_{33}^{*}=-\frac{v_{1}{}^{2}p_{32}+v_{1}(-c_{33}^{\left(2\right)}q_{12}-p_{32}+c_{33}^{\left(1\right)}q_{12})-c_{33}^{\left(1\right)}q_{12}}{q_{12}}\\ c_{44}^{*}=\frac{-2\kappa_{22}^{\varepsilon (1)}v_{1}e_{24}^{\left(2\right)}e_{24}^{\left(1\right)}q_{32}q_{42}+\left(2\kappa_{22}^{\varepsilon (1)}v_{1}q_{32}q_{42}+q_{32}{\left(q_{42}\right)}^{2}\right){\left(e_{24}^{\left(1\right)}\right)}^{2}}{q_{32}\kappa_{22}^{\varepsilon (1)}\kappa_{22}^{\varepsilon (2)}q_{42}}\\ \;+\frac{\kappa_{22}^{\varepsilon (1)}v_{1}{\left(e_{24}^{\left(2\right)}\right)}^{2}q_{32}q_{42}-{\left(c_{44}^{\left(2\right)}\kappa_{22}^{\varepsilon (1)}\right)}^{2}\kappa_{22}^{\varepsilon (1)}-c_{44}^{\left(2\right)}\kappa_{22}^{\varepsilon (2)}{\left(\kappa_{22}^{\varepsilon (1)}\right)}^{2}q_{32}}{q_{32}\kappa_{22}^{\varepsilon (1)}\kappa_{22}^{\varepsilon (2)}q_{42}}\\ \;+\frac{\left(-q_{32}{\left(q_{42}\right)}^{2}-\kappa_{22}^{\varepsilon (1)}v_{1}q_{32}q_{42}\right){\left(e_{24}^{\left(1\right)}\right)}^{2}+c_{44}^{\left(2\right)}c_{44}^{\left(1\right)}{\left(\kappa_{22}^{\varepsilon (2)}\right)}^{2}\kappa_{22}^{\varepsilon (1)}}{q_{32}\kappa_{22}^{\varepsilon (1)}\kappa_{22}^{\varepsilon (2)}q_{42}}\\ \;+\frac{v_{1}{}^{2}{\left(\kappa_{22}^{\varepsilon (1)}\right)}^{2}q_{32}p_{42}+c_{44}^{\left(2\right)}{\left(\kappa_{22}^{\varepsilon (2)}\right)}^{2}\kappa_{22}^{\varepsilon (1)}q_{32}-{\left(c_{44}^{\left(2\right)}\right)}^{2}q_{42}\kappa_{22}^{\varepsilon (1)}\kappa_{22}^{\varepsilon (2)}}{q_{32}\kappa_{22}^{\varepsilon (1)}\kappa_{22}^{\varepsilon (2)}q_{42}}\\ c_{55}^{*}=c_{55}^{\left(2\right)}v_{1}+c_{55}^{\left(1\right)}-c_{55}^{\left(1\right)}v_{1}\\ c_{66}^{*}=\frac{1}{2}\frac{\left(c_{22}^{\left(1\right)}-c_{12}^{\left(1\right)})(c_{22}^{\left(2\right)}-c_{12}^{\left(2\right)}\right)}{q_{22}-q_{12}} \end{array}.$$

where $v_{1}=a/(a+c)$ is volume fraction of the unit cell during the first homogenization, and the components of the matrix p and the matrix q are defined as

(A4) $$\begin{array}{l} p_{12}={\left(c_{12}^{\left(1\right)}-c_{12}^{\left(2\right)}\right)}^{2}\\ p_{22}=\left(c_{12}^{\left(1\right)}-c_{12}^{\left(2\right)}\right)\left(c_{23}^{\left(1\right)}-c_{23}^{\left(2\right)}\right)\\ p_{32}={\left(c_{23}^{\left(1\right)}-c_{23}^{\left(2\right)}\right)}^{2}\\ p_{42}={\left(e_{24}^{\left(1\right)}-e_{24}^{\left(2\right)}\right)}^{2}\\ p_{52}=\left(c_{23}^{\left(1\right)}-c_{23}^{\left(2\right)}\right)\left(e_{32}^{\left(1\right)}-e_{32}^{\left(2\right)}\right)\\ p_{62}=\left(c_{12}^{\left(1\right)}-c_{12}^{\left(2\right)}\right)\left(e_{32}^{\left(1\right)}-e_{32}^{\left(2\right)}\right)\\ p_{72}=-{\left(e_{32}^{\left(1\right)}-e_{32}^{\left(2\right)}\right)}^{2} \end{array}$$

and

(A5) $$\begin{array}{l} q_{12}=-v_{1}c_{22}^{\left(1\right)}-c_{22}^{\left(2\right)}+c_{22}^{\left(2\right)}v_{1}\\ q_{22}=-v_{1}c_{12}^{\left(1\right)}-c_{12}^{\left(2\right)}+c_{12}^{\left(2\right)}v_{1}\\ q_{32}=-v_{1}c_{44}^{\left(1\right)}-c_{44}^{\left(2\right)}+c_{44}^{\left(2\right)}v_{1}\\ q_{42}=-v_{1}\kappa_{22}^{\varepsilon (1)}-\kappa_{22}^{\varepsilon (2)}+\kappa_{22}^{\varepsilon (2)}v_{1} \end{array}$$

## Appendix B. Non-Zero Components of the Generalized Elasticity Tensors after the Second Homogenization

**(a) Piezoelectric Coefficients:**

(B1) $$\begin{array}{l} e_{24}^{\mathrm{Eff}}=e_{24}^{*}v_{2}+e_{24}^{\left(1\right)}-e_{24}^{\left(1\right)}v_{2}\\ e_{32}^{\mathrm{Eff}}=-\frac{-e_{32}^{\left(1\right)}q_{11}^{*}+v_{2}{}^{2}p_{61}^{*}-v_{2}p_{61}^{*}-v_{2}e_{32}^{*}q_{11}^{*}+v_{2}e_{32}^{\left(1\right)}q_{11}^{*}}{q_{11}^{*}}\\ e_{15}^{\mathrm{Eff}}=\frac{\left(-e_{15}^{*}+e_{15}^{\left(1\right)}\right)\kappa_{11}^{\varepsilon (1)}v_{2}+e_{15}^{\left(1\right)}q_{41}^{*}}{q_{41}^{*}}\\ e_{33}^{\mathrm{Eff}}=-\frac{v_{2}e_{33}^{\left(1\right)}q_{11}^{*}-v_{2}p_{51}^{*}-v_{2}e_{33}^{*}q_{11}^{*}+v_{2}{}^{2}p_{51}^{*}-e_{33}^{\left(1\right)}q_{11}^{*}}{q_{11}^{*}}\\ e_{31}^{\mathrm{Eff}}=-\frac{\left(e_{31}^{*}-e_{31}^{\left(1\right)}\right)c_{11}^{*}v_{2}+\left(-e_{31}^{*}+e_{31}^{\left(1\right)}\right)c_{11}^{*}-e_{31}^{*}q_{11}^{*}}{q_{11}^{*}} \end{array}$$

**(b) Relative Dielectric Constants:**

(B2) $$\begin{array}{l} \kappa_{11}^{\varepsilon -\mathrm{Eff}}=-\frac{\kappa_{11}^{\varepsilon *}\kappa_{11}^{\varepsilon (1)}}{q_{41}^{*}}\\ \kappa_{22}^{\varepsilon -\mathrm{Eff}}=\kappa_{22}^{\varepsilon *}v_{2}+\kappa_{22}^{\varepsilon (1)}-\kappa_{22}^{\varepsilon (1)}v_{2}\\ \kappa_{33}^{\varepsilon -\mathrm{Eff}}=\frac{-v_{2}\kappa_{33}^{\varepsilon (1)}q_{11}^{*}+v_{2}\kappa_{33}^{\varepsilon *}q_{11}^{*}-v_{2}{}^{2}p_{71}^{*}+\kappa_{33}^{\varepsilon (1)}q_{11}^{*}+v_{2}p_{71}^{*}}{q_{11}^{*}} \end{array}$$

**(c) Elastic Constants:**

(B3) $$\begin{array}{l} c_{11}^{\mathrm{Eff}}=-\frac{c_{11}^{*}c_{11}^{\left(1\right)}}{q_{11}^{*}}\\ c_{12}^{\mathrm{Eff}}=-\frac{(c_{12}^{*}-c_{12}^{\left(1\right)})c_{11}^{*}\left(v_{2}-1\right)-c_{12}^{*}q_{11}^{*}}{q_{11}^{*}}\\ c_{13}^{\mathrm{Eff}}=-\frac{(c_{13}^{*}-c_{13}^{\left(1\right)})c_{11}^{*}\left(v_{2}-1\right)-c_{13}^{*}q_{11}^{*}}{q_{11}^{*}}\\ c_{22}^{\mathrm{Eff}}=-\frac{v_{2}{}^{2}p_{11}+v_{2}(-c_{22}^{*}q_{11}^{*}-p_{11}^{*}+c_{22}^{\left(1\right)}q_{11}^{*})-c_{22}^{\left(1\right)}q_{11}^{*}}{q_{11}^{*}}\\ c_{23}^{\mathrm{Eff}}=-\frac{v_{2}{}^{2}p_{21}+v_{2}(-c_{23}^{*}q_{11}^{*}-p_{21}^{*}+c_{23}^{\left(1\right)}q_{11}^{*})-c_{23}^{\left(1\right)}q_{11}^{*}}{q_{11}^{*}}\\ c_{33}^{\mathrm{Eff}}=-\frac{v_{2}{}^{2}p_{31}+v_{2}(-c_{33}^{*}q_{11}^{*}-p_{31}^{*}+c_{33}^{\left(1\right)}q_{11}^{*})-c_{33}^{\left(1\right)}q_{11}^{*}}{q_{11}^{*}}\\ c_{44}^{\mathrm{Eff}}=c_{44}^{*}v_{2}+c_{44}^{\left(1\right)}-c_{44}^{\left(1\right)}v_{2}\\ c_{55}^{\mathrm{Eff}}=\frac{-2\kappa_{11}^{\varepsilon (1)}v_{2}e_{15}^{*}e_{15}^{\left(1\right)}q_{31}^{*}q_{41}^{*}+\left(2\kappa_{11}^{\varepsilon (1)}v_{2}q_{31}^{*}q_{41}^{*}+q_{31}^{*}{\left(q_{41}^{*}\right)}^{2}\right){\left(e_{15}^{\left(1\right)}\right)}^{2}}{q_{31}^{*}\kappa_{11}^{\varepsilon *}\kappa_{11}^{\varepsilon (1)}q_{41}^{*}}\\ \;+\frac{\kappa_{11}^{\varepsilon (1)}v_{2}{\left(e_{15}^{*}\right)}^{2}q_{31}^{*}q_{41}^{*}-{\left(c_{55}^{*}\kappa_{11}^{\varepsilon (1)}\right)}^{2}\kappa_{11}^{\varepsilon *}-c_{55}^{*}\kappa_{11}^{\varepsilon *}{\left(\kappa_{11}^{\varepsilon (1)}\right)}^{2}q_{31}^{*}}{q_{31}^{*}\kappa_{11}^{\varepsilon *}\kappa_{11}^{\varepsilon (1)}q_{41}^{*}}\\ \;+\frac{\left(-q_{31}^{*}{\left(q_{41}^{*}\right)}^{2}-\kappa_{11}^{\varepsilon (1)}v_{2}q_{31}^{*}q_{41}^{*}\right){\left(e_{15}^{\left(1\right)}\right)}^{2}+c_{55}^{*}c_{55}^{\left(1\right)}{\left(\kappa_{11}^{\varepsilon *}\right)}^{2}\kappa_{11}^{\varepsilon (1)}}{q_{31}^{*}\kappa_{11}^{\varepsilon *}\kappa_{11}^{\varepsilon (1)}q_{41}^{*}}\\ \;+\frac{v_{2}{}^{2}{\left(\kappa_{11}^{\varepsilon (1)}\right)}^{2}q_{31}^{*}p_{41}^{*}+c_{55}^{*}{\left(\kappa_{11}^{\varepsilon *}\right)}^{2}\kappa_{11}^{\varepsilon (1)}q_{31}^{*}-{\left(c_{55}^{*}\right)}^{2}q_{41}^{*}\kappa_{11}^{\varepsilon (1)}\kappa_{11}^{\varepsilon *}}{q_{31}^{*}\kappa_{11}^{\varepsilon *}\kappa_{11}^{\varepsilon (1)}q_{41}^{*}}\\ c_{66}^{\mathrm{Eff}}=\frac{1}{2}\frac{\left(c_{11}^{*}-c_{12}^{*})(c_{11}^{\left(1\right)}-c_{12}^{\left(1\right)}\right)}{q_{21}^{*}-q_{11}^{*}} \end{array}$$

where $v_{2}=b/(b+d)$ is the volume fraction of the unit cell during the second homogenization, and the components of the matrix $p^{*}$ and the matrix $q^{*}$ are defined as

(B4) $$\begin{array}{l} p_{11}^{*}={\left(c_{12}^{*}-c_{12}^{\left(1\right)}\right)}^{2}\\ p_{21}^{*}=\left(c_{12}^{*}-c_{12}^{\left(1\right)}\right)\left(c_{13}^{*}-c_{13}^{\left(1\right)}\right)\\ p_{31}^{*}={\left(c_{13}^{*}-c_{13}^{\left(1\right)}\right)}^{2}\\ p_{41}^{*}={\left(e_{15}^{*}-e_{15}^{\left(1\right)}\right)}^{2}\\ p_{51}^{*}=\left(c_{13}^{*}-c_{13}^{\left(1\right)}\right)\left(e_{31}^{*}-e_{31}^{\left(1\right)}\right)\\ p_{61}^{*}=\left(c_{12}^{*}-c_{12}^{\left(1\right)}\right)\left(e_{31}^{*}-e_{31}^{\left(1\right)}\right)\\ p_{71}^{*}=-{\left(e_{31}^{*}-e_{31}^{\left(1\right)}\right)}^{2} \end{array}$$

and

(B5) $$\begin{array}{l} q_{11}^{*}=-v_{2}c_{11}^{\left(1\right)}-c_{11}^{*}+c_{11}^{*}v_{2}\\ q_{21}^{*}=-v_{2}c_{12}^{\left(1\right)}-c_{12}^{*}+c_{12}^{*}v_{2}\\ q_{31}^{*}=-v_{2}c_{55}^{\left(1\right)}-c_{55}^{*}+c_{55}^{*}v_{2}\\ q_{41}^{*}=-v_{2}\kappa_{11}^{\varepsilon (1)}-\kappa_{11}^{\varepsilon *}+\kappa_{11}^{\varepsilon *}v_{2} \end{array}$$
